# LINE retrotransposition and host DNA repair machinery

**DOI:** 10.1080/2159256X.2015.1096998

**Published:** 2015-10-20

**Authors:** Katsumi Yamaguchi, Masaki Kajikawa, Norihiro Okada

**Affiliations:** 1 Graduate School of Bioscience and Biotechnology; Tokyo Institute of Technology; Yokohama, Kanagawa Japan; 2 Department of Life Sciences; National Cheng Kung University; Tainan, Taiwan; 3 Foundation for Advancement of International Science; Tsukuba, Japan

**Keywords:** DNA double-strand breaks, long interspersed element, microhomology-mediated end joining, non-homologous end joining, Non-LTR retrotransposon, retrotransposition

## Abstract

Long interspersed elements (LINEs), or non-long-terminal repeat (LTR) retrotransposons, are mobile genetic elements that exist in the genomic DNA of most eukaryotes, comprising a considerable portion of the host chromosomes. LINEs constitute endogenous mutagens that cause insertional mutations in host chromosomes and have a large impact on host genome evolution. Despite their importance, however, the molecular mechanism of LINE retrotransposition is not fully understood. Several studies suggest that host proteins that participate in the repair of DNA breaks modulate LINE retrotransposition. Recently, we provided evidence that there are 2 distinct pathways—annealing and direct—that join the 5′-end of LINEs to host chromosomal DNA. These pathways appear to be used distinctively by zebrafish LINEs and the human L1 in DT40 cells. In HeLa cells, only the annealing pathway appears to be used. This implies that different characteristics of the 2 LINEs and also host factors dictate which pathway is selected. Here, we discuss the 5′-end-joining pathways of LINE retrotransposition and propose that the pathways of LINE integration adopt certain host repair factors.

## Introduction

Long interspersed elements (LINEs), or non-long-terminal repeat (LTR) retrotransposons, are mobile genetic elements that exist in the genomic DNA of most eukaryotes. LINEs mobilize and amplify their own copies via retrotransposition. Thus, LINEs constitute an endogenous mutagen that causes insertional mutations of LINEs in the host genome. Moreover, a huge number of LINE copies are accumulated in the genome of many eukaryotes during evolution, comprising a considerable portion of the host chromosome. The human genome, for example, contains ∼850,000 copies of LINEs, accounting for ∼20% of the genome.^1^ The successful expansion of LINEs in eukaryotic genomes and many experimental data indicate that LINEs have a large impact on genome evolution.^2,3^ However, the molecular mechanism of LINE retrotransposition is not well understood.

Typical LINEs encode 2 proteins, called open-reading frame 1 and 2 (ORF1p and ORF2p), which are involved in their own retrotransposition. ORF1p possesses the LINE's nucleic acid–binding and nucleic acid chaperone activities,^4-6^ and ORF2p possesses the endonuclease and reverse transcriptase (RT) activities.^7,8^ During retrotransposition, the LINE RNA is initially transcribed from the LINE DNA and is transported to the cytoplasm where the LINE-encoded proteins are translated. Next, the LINE RNA and proteins form an RNA-protein complex, most likely through the nucleic acid–binding activity of ORF1p.^5^ The RNA-protein complex then moves back to the nucleus where the endonuclease of ORF2p nicks a single strand of the host genomic DNA at the target site, thereby generating a 3′ hydroxyl group.^9,10^ The RT of ORF2p utilizes the single 3′ hydroxyl group as a primer to initiate reverse transcription of the LINE RNA. This reaction is a characteristic feature of LINE retrotransposition and is called target-primed reverse transcription (TPRT).^11,12^ The nucleic acid chaperone activity of ORF1p has been proposed to help the annealing of the LINE RNA and the primer strand of the genomic DNA to initiate reverse transcription, although its precise role in retrotransposition is not clear.^13,14^ TPRT connects the 3′-end of the newly synthesized LINE DNA to the host genomic DNA, yet at this stage the LINE remains as a DNA/RNA hybrid. The molecular mechanism of LINE retrotransposition after TPRT has not been well documented. In particular, the molecular basis of 5′-end joining of the newly synthesized LINE to genomic DNA has not been well elucidated. Seemingly, the nucleic acids of the target site after TPRT must form a branched structure that does not exist in the intact chromosome, suggesting that it is recognized as a DNA break by the host repair system. Also, it is noteworthy that LINEs do not encode any proteins homologous to host repair proteins, suggesting that host DNA repair pathways or proteins participate in LINE retrotransposition. Indeed, recent studies suggest that several host proteins that participate in DNA repair are also involved in retrotransposition.^15-18^ Here, we discuss the host DNA repair machinery that participates in LINE mobilization.

## Host DNA Repair Proteins Participate in LINE Retrotransposition

Eukaryotes encode a large number of proteins involved in DNA repair to maintain genomic integrity. DNA double-strand breaks (DSBs) are one of the most deleterious forms of damage for a host, and such breaks must be repaired immediately. Eukaryotes have 2 predominant pathways for DSB repair: homologous recombination and non-homologous end joining (NHEJ). Homologous recombination repairs DSBs using homologous chromosomes, and thus the repaired junction retains the original sequence. NHEJ, however, directly connects the 2 broken ends without utilizing any genetic information of homologous chromosomes, resulting in altered DNA sequences at the repaired junction. NHEJ requires several host proteins, such as Ku70/80, DNA-PKcs, Artemis, XRCC4, XRF, and Ligase IV.^19^ Interestingly, NHEJ can be detected in vivo and in vitro even if Ku70/80 or Ligase IV is defective, indicating that there is an alternate NHEJ pathway(s) that does not require these 2 core proteins.^20,21^ To avoid confusion, NHEJ that depends on the core proteins is termed classical NHEJ (C-NHEJ), whereas the core protein–independent pathway is termed alternative NHEJ (alt-NHEJ).^22^ Although the proteins responsible for alt-NHEJ are not fully understood, a few proteins such as PARP, polymerase θ, and Ligase III are proposed to be involved.^23-25^ The physiological importance of alt-NHEJ remains to be elucidated, and it is unclear whether alt-NHEJ is a single definitive pathway or an assemblage of multiple pathways.^22^ Recently, microhomology-mediated end joining (MMEJ) was defined as an alt-NHEJ pathway.^22^

Several host repair proteins have been shown to participate in LINE retrotransposition. Deficiency of ataxia telangiectasia mutated (ATM), a key sensor protein kinase of various cell stresses including DSBs, influences the retrotransposition frequency of the human LINE, L1, in cultured cells and in mice.^15,18^ The role of ATM in L1 retrotransposition is, however, controversial because 2 groups have reported opposing results; one showed that ATM deficiency increases L1 retrotransposition frequency,^15^ whereas the other showed it decreases the frequency.^18,26^ This discrepancy may reflect that ATM is a versatile protein that induces many responses against various cell stresses,^27^ and thus, the role of ATM in L1 retrotransposition might be complicated. A flap endonuclease, ERCC1/XPF, also modulates L1 retrotransposition.^16^ ERCC1/XPF deficiency in human cells raises L1 retrotransposition frequency, suggesting that this endonuclease limits L1 retrotransposition. Because ERCC1/XPF is a structure-specific endonuclease that cleaves the junction of single- to double-stranded DNAs, the nuclease may cut the branching DNA at the target site, resulting in the elimination of the LINE.^16^ Previously, we showed that the 4 core proteins of C-NHEJ (Ku70, DNA-PKcs, Artemis, and Ligase IV) are involved in LINE retrotransposition in human HeLa-RC or chicken DT40 cells, probably participating in the 5′-end joining of LINEs.^17^ This body of evidence suggests that several host repair proteins participate in LINE retrotransposition, although the role of the host repair machinery in retrotransposition is not fully understood.

## 5′-End Joining of LINEs and Host Repair Machinery in DT40 Cells

**Figure 1.**
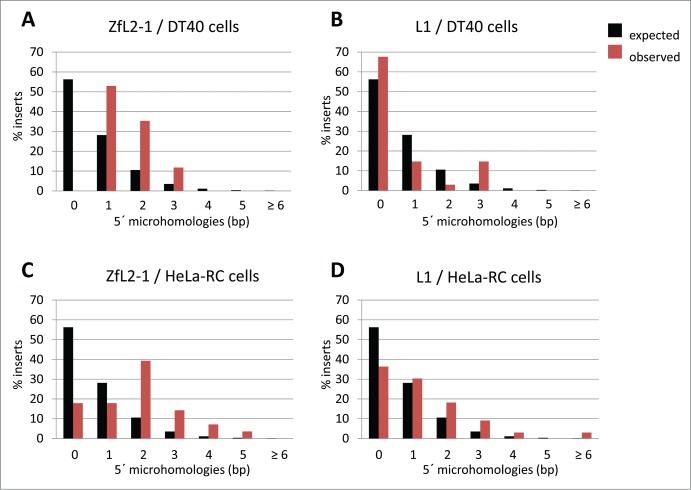
Length distribution of microhomologies at the 5´-end junctions of LINE integrants retrotransposed in cultured cells. Black bars represent the expected length distribution of microhomologies described by Roth et al. (1985).^39^ Red bars represent the observed length distribution of microhomologies at the 5´-end junctions of LINE integrants. (**A**) ZfL2–1 integrants in DT40 cells. (**B**) L1 integrants in DT40 cells. (**C**) ZfL2–1 integrants in HeLa-RC cells. (**D**) L1 integrants in HeLa-RC cells.

**Figure 2.**
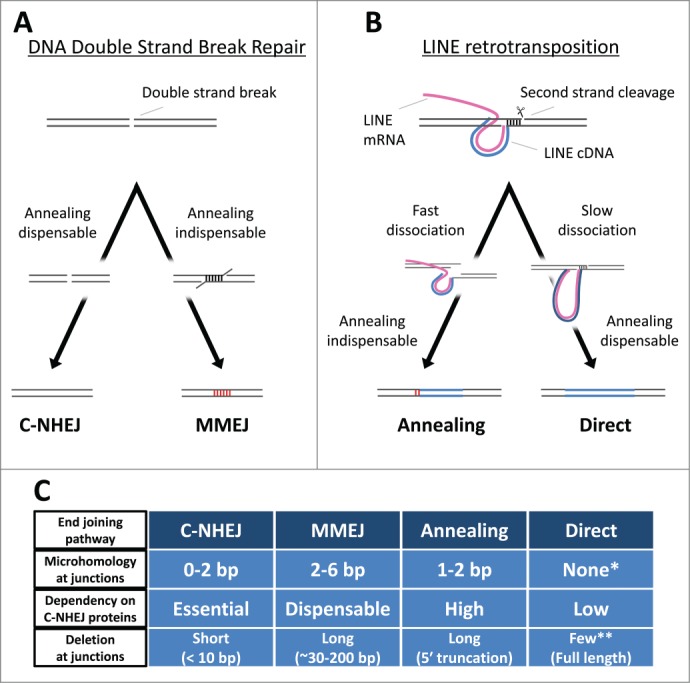
The end-joining pathways. (**A**) The 2 non-homologous end-joining pathways. C-NHEJ and MMEJ exhibit different requirements for host repair proteins, yielding distinctive repaired junctions (see text for details). Blue lines, LINE DNA. Magenta lines, LINE RNA. Red vertical lines, microhomologies. (**B**) The 2 5′-end-joining pathways of LINEs. There are at least 2 distinct end-joining pathways, Annealing and Direct, which connect the 5′-ends of LINEs to host genomic DNA (see text for details). (**C**) The characteristics of the 4 end-joining pathways. The two annealing-dispensable pathways, C-NHEJ and Direct, can join the 2 genomic ends with no microhomology (MH) and usually does not yield long deletion at the junctions. The two pathways are, however, distinct from each other in respect of the dependency on the C-NHEJ proteins. The two annealing-indispensable pathways, MMEJ and Annealing, certainly accompany MH and frequently yield long deletions at the junctions. The two pathways are also distinct from each other in respect of the dependency on the C-NHEJ proteins (see text for details). *The length distribution of microhomologies with the direct end-joining pathway is almost the same as that expected (**Fig. 1B**). **The direct end-joining pathway predominantly produces full-length integrants with no 5′ truncation^31^.

The sequence characteristics at the fixed junction of DSBs reflect which end-joining pathway is involved in repairing the breaks. Recent studies of chromosomal rearrangement in human cells clearly demonstrate that C-NHEJ and MMEJ predominantly accompany 0–2 bp and 2–6 bp of microhomology (MH) at the junction, respectively.^28,29^ Also, the 2 pathways cause distinct lengths of genomic deletions at the repaired junctions; C-NHEJ typically generates deletions shorter than 10 bp, whereas MMEJ generates deletions of ∼30–200 bp.^28,29^ Similarly, analysis of the features of the 5′-end junctions of LINE integrants makes it possible for us to infer which kind of DNA repair pathway is involved in their 5′-end joining. The 5′ junctions of LINE integrants in DT40 cells can be classified into 3 types: junctions with MH, junctions with extra nucleotides (EX), and junctions without MH or EX (we refer to this type as direct joining).^30,31^ In the case of integrants of zebrafish LINEs (ZfL2–1 and ZfL2–2) in DT40 cells, roughly half have ∼1–2 bp of MH (**Fig. 1A**), and the other half have EX; those with direct joining are rare.^17,31^ Sequence analysis of EX to deduce its origin suggests that genomic DNA and/or the LINE DNA flanking the junction is used as a template, and the synthesis likely occurs via annealing between the ends of the genomic and LINE DNAs during 5′-end joining.^30^ Similarly, MH at a repaired junction of DSBs is considered to be generated from annealing during the end-joining reaction (**Fig. 2A**).^22^ Thus, the 5′ junctions of the zebrafish LINEs in DT40 cells appear to be ligated by an annealing-mediated end-joining pathway (**Fig. 2B**). Although the molecular basis of this pathway may resemble that of MMEJ in which an annealing step must also occur (**Fig. 2A**), these pathways are most likely distinct because the features of the junctions connected by these 2 pathways are different (**Fig. 2C**). For example, the length of MH at the 5′ junctions of the zebrafish LINEs is shorter (generally 1–2 bp) than that of MMEJ (2–6 bp) (**Fig. 2C**), implying that the 5′-end joining of the zebrafish LINEs in DT40 cells is through a previously undefined pathway that is distinct from MMEJ. In contrast, integrants of human L1 in DT40 do not accompany MH or EX at their 5′ junctions (**Fig. 1B**),^31^ indicating that a molecular mechanism including an annealing step is not involved in the 5′-end joining of human L1 in these cells (**Fig. 2B, C**). The absence of MH apparently indicates that the 5′-end joining of human L1 occurs through C-NHEJ. However, L1 retrotransposition in DT40 cells is not exclusively dependent on the C-NHEJ core proteins.^17^ Thus, the pathway responsible for the 5′-end joining of L1 in DT40 cells also appears to be distinct from C-NHEJ and MMEJ (**Fig. 2C**). The retrotransposition of zebrafish ZfL2–2 in DT40 cells, however, depends mainly on the C-NHEJ core proteins,^17^ further supporting the idea that the annealing-mediated pathway to join the 5′-end of the zebrafish LINEs is distinct from MMEJ (**Fig. 2C**). To distinguish the 2 5′-end-joining pathways of LINEs in DT40 cells, we refer to the pathway of the zebrafish LINEs with MH or EX as ‘annealing’ and that of the human LINE without MH or EX as ‘direct’.^31^ Together, our studies suggest that even for DT40 cells there are 2 distinct pathways to connect the 5′-ends of LINEs to host chromosomal DNA, which are most likely distinct from both C-NHEJ and MMEJ and are previously unrecognized (**Fig. 2**). This is feasible because the intermediate branched structure of LINE retrotransposition generated by TPRT does not occur with normal DNA breaks. The putative novel end-joining pathways may exist to specifically repair the breaks induced by LINE retrotransposition. Because LINEs have settled in the genomic DNA of most eukaryotes in a long time ago,^32^ it is reasonable to speculate that host organisms have developed specific combinations of repair factors (or specific systems) to repair LINE-induced breaks.

## How Are the 5′-End-Joining Pathways Selected in DT40 cells?

The proposed end-joining pathways—annealing and direct—are exclusively used to join the 5′-ends of ZfL2–1 and L1, respectively, in DT40 cells (**Fig. 2B**).^31^ This implies that distinct characteristics between ZfL2–1 and L1 dictate which pathway is used. The second strand of the target site must be cleaved so that the newly synthesized LINE DNA is inserted into the host chromosome. Although the nuclease responsible for the second-strand cleavage has not been definitively determined, the endonucleases encoded by a few site-specific LINEs are shown to cleave both the first and second strands of their target sites.^9,33^ Regardless of which nuclease cuts the second strand, the relative position of the first- and second-strand cleavages can be deduced from the sequence of target-site alteration.^31,34^ Analysis of the target-site alteration suggests that the cleaved position of the second strand against that of the first strand is uniquely dependent on each LINE.^31,35^ For example, L1 cuts the second strand of the target site ∼15 bp downstream of the first-strand nick, whereas ZfL2–1 cuts ∼5 bp downstream.^31^ The relative position of the first- and second-strand cleavages determines which kind of the genomic DNA end is generated. In the case of L1, the genomic end created at the target site must have a 3′ overhang of ∼15 nt, whereas, in the case of ZfL2–1, the end is created with a 3′ overhang of ∼5 nt. At the time of second-strand cleavage, the 3′ overhang probably forms a duplex with its complementary genomic strand, keeping the 2 genomic ends together at the target site. However, they must be dissociated for the newly synthesized LINE DNA to be inserted. Our study suggests that the extent of dissociation of the genomic synapsis is distinct depending on the length of the 3′ overhang; the genomic synapsis of L1 through the ∼15 bp duplex is more stable than that of ZfL2–1 through the ∼5 bp duplex.^31^ Because the distinct difference of stability appears to dictate the structure of the retrotransposition intermediate that must be recognized and repaired by the host repair machinery, we speculate that, to join their 5′-ends with the genomic DNA, the slow dissociation of L1 allows recruitment of components of the direct end-joining pathway, whereas the fast dissociation of ZfL2–1 recruits the annealing pathway(**Fig. 2B**).

## Molecular Basis of the LINE 5′ Truncation

Most LINEs in the host genome have a truncation of a certain length at their 5′-ends, called 5′ truncation. LINEs with 5′ truncation are variable in length and are usually retrotransposition incompetent because they are missing sequences essential for retrotransposition, such as the internal promoter and protein-coding regions. Although the molecular basis for 5′ truncation is not known, it is generally assumed to be caused by the low processivity of the LINE RT.^36^ Recently, one paper proposed that ATM modulates the length of L1 integrants in human cells,^18^ but the mechanism has not been reported. We previously showed that deficiency of one of the C-NHEJ core proteins Ku70, Artemis, or ligase IV results in the production of longer integrants of the zebrafish LINE ZfL2–2 in DT40 cells, indicating that these proteins limit the LINE length.^17^ In the same paper, we suggested that the C-NHEJ factors, especially Ku70/80, recognize the retrotransposition intermediate that is under reverse transcription of the LINE RNA as a DNA break and thus join the 5′-end of the LINE DNA to the host genomic DNA before reverse transcription is completed. Therefore, the 5′ truncation of LINEs is probably generated by the action of host repair proteins rather than as a consequence of the low processivity of the RT. Even after reverse transcription has been completed, the annealing-mediated end-joining pathway may be able to produce a 5′ truncation through annealing at the internal region of the LINE DNA to the genomic DNA end. That is, the LINE DNA upstream of the annealing duplex may be trimmed by the end-joining pathway. In contrast to the 5′ region of the LINE DNA, the genomic DNA is not largely deleted in general in retrotransposition. This is in contrast to MMEJ, through which the genomic DNA flanking the break point is substantially deleted during repair,^28,29^ supporting again the idea that the annealing-mediated end-joining pathway of the zebrafish LINEs in DT40 cells is distinct from MMEJ. Although the host repair proteins are required for LINE retrotransposition, they seem to concomitantly exert the effect to limit further retrotransposition through the production of LINEs with a 5′ truncation, which cannot mobilize.^17^ Interestingly, L1 produces a large number of full-length elements with no 5′ truncation in DT40 cells, whereas ZfL2–1 rarely produces full-length elements.^31^ The difference in the ability to yield full-length elements is consistent with the hypothesis that each LINE selects one of the 2 end-joining pathways—annealing or direct. In particular, the ∼15-bp genomic synapsis generated during the 5′-end joining of L1 is stable enough to complete reverse transcription, resulting in the generation of a large number of full-length elements (**Fig. 2B****, slow dissociation**). In contrast, the relatively unstable genomic synapsis of ∼5 bp generated during the 5′-end joining of ZfL2–1 does not provide sufficient time to yield full-length elements (**Fig. 2B**, **fast dissociation**). Thus, L1 might be able to escape the limiting effect of host repair proteins when retrotransposing in DT40 cells. Taken together, we speculate that the stability of the genomic synapsis of a duplex whose length is unique in each LINE dictates which 5′-end-joining pathway is selected in DT40 cells, affecting the generation of 5′ truncation.

## Generality of the 5′-End-Joining Pathways

Several studies suggest that annealing and direct end-joining pathways are also present in cells other than DT40, such as human cells,^31,37,38^ indicating the generality of these pathways. However, the extent of their participation in the 5′-end joining of LINEs appears to be governed by cell type as well as LINE species.^30,31^ Remarkably, the L1 integrants retrotransposed in HeLa-RC cells predominantly have MH of ∼1–2 bp at their 5′ junctions (**Fig. 1D**), in contrast to those in DT40 cells, which predominantly have no MH (**Fig. 1B**).^31^ Also, the ZfL2–1 integrants in HeLa-RC cells mainly have MH of ∼1–2 bp (**Fig. 1C**). The fact that both L1 and ZfL2–1 mostly have MH at their 5′ junctions in HeLa-RC cells suggests that the annealing-mediated end joining is the predominant pathway regardless of LINE species in HeLa-RC cells (**Fig. 1C, D**). In other words, the length of the 3′ overhang generated at the target site by the first- and second-strand cleavages does not appear to dictate which end-joining pathway works for each LINE in HeLa-RC cells. In these cells, the genomic synapsis through the 3′ overhang might be disassembled immediately after the second-strand cleavage regardless of its length, resulting in the initiation of the annealing-mediated end-joining pathway. Host proteins, rather than the characteristics of LINEs, are therefore the main determinants for the annealing-mediated end-joining pathway in HeLa-RC cells. Indeed, L1 as well as ZfL2–1 and ZfL2–2 rarely produce full-length integrants in HeLa-RC cells,^31^ supporting the idea that the generation of a 5′ truncation is controlled by host repair proteins rather than the characteristics of the LINE RT itself.

The occurrence of EX also varies depending on cell type and LINE species. Notably, ZfL2–1 frequently yields EX when retrotransposing in DT40 cells but not in HeLa-RC cells. This suggests that, although ZfL2–1 appears to mobilize through the annealing-mediated end-joining pathway in both cell types, the pathways are somewhat distinct between these cell types. We previously provided evidence that, in DT40 cells, a large proportion of the EX of ZfL2–2 inserts is synthesized using flanking sequences as a template. Accordingly, a DNA polymerase might be involved in EX synthesis, and its activity may be greater during LINE retrotransposition in DT40 cells than in HeLa-RC cells, explaining the observed greater occurrence of EX in DT40 cells.

In contrast, a comparable analysis of target-site alteration in HeLa-RC and DT40 cells suggests that a nuclease activity predominantly works during LINE retrotransposition in HeLa-RC cells.^31^ Polymerases and nucleases have important roles in DSB repair—they make the broken ends competent for ligation. Our observation may indicate that the mechanism by which broken DNA ends are processed to become ligation competent can be classified into 2 types, namely polymerase-predominant and nuclease-predominant, at least in the case of the 5′-end joining of LINEs. In summary, recent studies suggest that there are at least 2 distinct pathways—annealing and direct—that connect the 5′-end of LINEs to host genomic DNA, and those pathways are chosen based on both cell type and LINE species.

## Future Perspectives

Recent studies indicate that host DNA repair machinery is involved in LINE retrotransposition. Although several host repair proteins are involved in LINE retrotransposition, many other proteins are likely involved, most of which have not been identified. The next work important for elucidating the molecular mechanism of LINE retrotransposition is to identify which and how many host DNA repair proteins are involved. It is also important to identify the host repair proteins essential for each of the annealing and direct end-joining pathways. Future work should be focused not only on the elucidation of the molecular basis of LINE retrotransposition but also on a more comprehensive understanding of the host DNA repair machineries and their relationships with those of LINE retrotransposition.
